# Polymorphisms of KLF3 gene coding region and identification of their functionality for abdominal fat in chickens

**DOI:** 10.1002/vms3.422

**Published:** 2020-12-27

**Authors:** Weijia Wang, Yudong Li, Ziwei Li, Ning Wang, Fan Xiao, Haihe Gao, Huaishun Guo, Hui Li, Shouzhi Wang

**Affiliations:** ^1^ Key Laboratory of Chicken Genetics and Breeding Ministry of Agriculture and Rural Affairs Harbin China; ^2^ Key Laboratory of Animal Genetics, Breeding and Reproduction Education Department of Heilongjiang Province Harbin China; ^3^ College of Animal Science and Technology Northeast Agricultural University Harbin China; ^4^ Fujian Sunnzer Biotechnology Development Co., Ltd. Guangze Fujian Province China

**Keywords:** abdominal fat, chicken, functional variants, Kruppel‐like factor 3, reporter gene assay

## Abstract

KLF3 is a member of the Kruppel‐like factor (KLF) family of transcription factors, and plays an important role in several biological processes, including adipogenesis, erythropoiesis and B‐cell development. The purposes of this study are to search for polymorphisms of *KLF3* coding region and to provide functional evidence for abdominal fat in chickens. A total of 168 SNPs in *KLF3* coding region were detected in a unique chicken population, the Northeast Agricultural University broiler lines divergently selected for abdominal fat content (NEAUHLF). Of which three single nucleotide polymorphisms (g.3452T > C, g.8663A > G and g.10751G > A) were significantly correlated with abdominal fat weight (AFW) and abdominal fat percentage (AFP) of 329 birds from the 19th generation of NEAUHLF (FDR < 0.05). The reporter gene assay was performed to verify functionality of these three SNPs in both ICP‐1 and DF1 cells. Results showed that the luciferase activity of G allele was significantly higher than that of A allele in g.10751G > A (*p* < 0.05). However, there were no significant differences between different alleles of others two SNPs in luciferase activity. Overall, *KLF3* is an important candidate gene that affects chicken abdominal fat content, and the g.10751G > A is a functional variant that potential would be applied to marker‐assisted selection (MAS) for selective breeding programme.

## Graphical abstract

A total of 168 SNPs in KLF3 coding region were detected in a unique chicken population. Three single nucleotide polymorphisms (g.3452T > C, g.8663A > G and g.10751G > A) were significantly correlated with abdominal fat. The reporter gene assay confirmed that g.10751G > A was a functional variant in both ICP‐1 and DF1 cells.

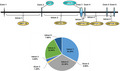

## 1 INTRODUCTION

With the rapid development of broiler industry, the problem of excessive accumulation of broiler body fat (especially abdominal fat) is becoming increasingly serious. Excessive fat deposition in broilers has adverse effects on feed conversion, carcass yield, hatching rate and fertility rate (Chen et al., 2019). Therefore, controlling the excessive accumulation of fat in chickens and further improving the feed conversion efficiency and carcass quality of broilers are important problems that need to be studied and solved urgently (Demeure et al., 2013; Zhang et al., 2018). Identification of functional variations associated with abdominal fat deposition in chickens is helpful for implementation of marker‐assisted selection (MAS) of abdominal fat traits and understanding of the genetic mechanism underlying fat growth and development.

Kruppel‐like factors (KLF) is a class of transcription factors with zinc finger structure, and widely involved in the regulation of cell proliferation, apoptosis, differentiation and embryonic development, and other life activities (Suske et al., 2005). KLF3, a member of the KLF family, is involved in the regulation of adipogenesis and lipid metabolism (Pearson et al., 2011). In vitro, *KLF3* is regarded as a repressor that depresses the core gene CCAAT‐enhancer binding protein alpha (CEBPα) in 3T3‐L1 adipogenesis. In vivo, *KLF3* knockout mice have smaller and fewer adipocytes and are leaner than wild‐type mice (Guo, Khan, et al., 2018; Sue et al., 2008).

A lot of studies showed that *KLF3* also plays an important role in adipogenesis and lipid metabolism in agricultural animals. The transcriptional activity of *KLF3* is promoted by KLF15 in bovine adipocytes (Guo, Khan, et al., 2018). Polymorphisms in transcription factor binding sites of the *KLF3* promoter region affect intramuscular fat content in Qinchuan cattle (Guo, Raza, et al., 2018). Our previous studies found that chicken *KLF3* is expressed in chicken adipose tissue, and regulates important genes involved in adipogenesis and lipid metabolism, more specifically, *KLF3* overexpression suppress chicken CCAAT/enhancer binding protein alpha (C/EBPα), fatty acid binding protein 4 (FABP4), fatty acid synthase (FASN) and lipoprotein lipase (LPL) promoter activities, but increase chicken peroxisome proliferator‐activated receptor gamma (PPARγ) promoter activity (Zhang et al., 2014).

Based on the function of *KLF3*, it may be an important gene influencing the chicken abdominal fat content. To date, however, the relationship between sequence variants and chicken abdominal fat traits remains unclear. The purposes of this study are to analyse the polymorphisms in the coding region of *KLF3*, to investigate the association of SNP markers with abdominal fat traits and to identify their functionality.

## 2 MATERIALS AND METHODS

### 2.1 Experimental populations and phenotype measurements

The Northeast Agricultural University broiler lines divergently selected for abdominal fat content (NEAUHLF) have been established since 1996, using abdominal fat percentage (AFP) [AFP = abdominal fat weight (AFW)/body weight at 7 weeks of age (BW_7_)] and plasma very low‐density lipoprotein (VLDL) levels as selection criteria (Guo et al., 2011; Leng et al., 2009). A total of 329 male birds (159 fat birds and 170 lean birds) from the 19th generation of NEAUHLF were used in this study. All male birds were slaughtered at 7 weeks of age. The blood was collected from wing veins before slaughtered and genomic DNA was extracted according to a procedure based on phenol/chloroform extraction and ethanol precipitation and properly kept (Leng et al., 2009). The body weight at 7 weeks of age (BW_7_) was measured before slaughtered and AFW were measured after slaughter. Abdominal fat percentage was calculated as the ratio of AFW to BW_7_ (AFP = AFW/BW_7_).

### 2.2 Single nucleotide polymorphism (SNP) detection

We constructed individual 350bp DNA libraries for 329 male birds of 19th‐generation population in NEAUHLF, and conducted whole‐genome sequencing. After alignment, variant calling was performed for all samples by using the Unified Genotyper function in GATK 3.3 software. SNPs were selected by using the Variant Filtration parameter in GATK (Zhang et al., 2020). A total of 168 SNPs were detected in the coding region of *KLF3*. Genotypes of these SNPs were available by sequencing of 329 birds.

### 2.3 Cell culture

Two cell lines were utilized for promoter luciferase reporter assay, one is DF1 cell line that is widely used for cellular and molecular studies in chickens (Mannstadt et al., 2008; Wang et al., 2013) and the other is an immortalized chicken pre‐adipocyte cell line (ICP‐1) from our laboratory (W. Wang et al., 2017).

Cells were cultured in DMEM/F12 medium (Gibco, New York, NY, USA) supplemented with 10% fetal bovine serum (BI, Germany), 100 units/ml penicillin and 100 mg/ml streptomycin, and incubated at 37^◦^C, 5% CO_2_.

### 2.4 Construction of promoter luciferase reporter gene vector

According to the chicken *KLF3* of GenBank (NC_006091.4), the DNA fragments containing SNPs loci were synthesized, and the DNA fragments of different alleles of *KLF3* g.3452T > C (SNP‐74), g.8663A > G (SNP‐133) and g.10751G > A (SNP‐154) were connected to the upstream of the pGL3‐promoter vector SV40, respectively, named pGL3‐74‐TT, pGL3‐74‐CC; pGL3‐133‐AA, pGL3‐133‐GG; pGL3‐154‐GG and pGL3‐154‐AA.

### 2.5 Transfection and activity detection of dual luciferase reporter vectors

For transfection of the luciferase reporter plasmid, cells were seeded in 24‐well plates, at 70%–80% confluence were washed with PBS, transfected with pGL3‐Promoter vector containing the SNP and pRL‐TK Renilla luciferase vector (Promega) as an internal control using Lipofectamine 2000 (Invitrogen, Carlsbad, CA, USA). After 48h of transfection, cell lysates were collected and operated according to the instructions of Promega's double luciferase detection kit. Firefly luciferase activity was normalized to Renilla luciferase.

### 2.6 Bioinformatics analysis

In order to investigate potential molecular mechanism underlying the association of abdominal fat content with chicken *KLF3* gene g.10751G > A, we carried out in silico analysis of the transcription factor binding site using three bioinformatic tools, including JASPAR (http://jaspar.binf.ku.dk/), TFBIND (http://tfbind.hgc.jp/) and Mulan (http://mulan.dcode.org/).

### 2.7 Statistical analysis

Allele frequencies were calculated and the difference of allele frequencies between the lean and fat lines was analysed by Chi‐square test. A value of *p* < 0.05 was used as the significant difference between the lean and fat lines. Population parameters, including observed heterozygosity (Ho), gene heterozygosity (He), effective allele numbers (Ne) and polymorphism information content (PIC), were calculated by Nei's methods through the MSR call online platform (http://www.msrcall.com).

The associations between the SNPs and abdominal fat content (AFW and AFP) were analysed by using JMP 11.2 software (SAS Institute, Cary, NC, USA). The models used were as follows:

$$Y=\mu +\text{Sire}\left(\text{Line}\right)+\text{Dam}\left(\text{Line},\text{Sire}\right)+G+\text{Line}+{\text{BW}}_{7}+e$$

Where Y was the dependent variable for the traits measured in the population (AFW and AFP), μ was the overall population mean of the traits, G was the genotype fixed effect, Sire was the gender fixed effect, Line was the Line fixed effect, BW at 7 weeks (BW_7_) as a linear covariate (except for the AFP% traits), e was the residual random error, Sire (Line) was the random effect of Sire nested within Line and Dam (Line, Sire) was the random effect of Dam nested within Line and Sire. FDR was calculated from the distribution of *p*‐values for the 168 SNPs, and FDR < 0.05 was considered as statistically significant for association (Matsuoka et al., 2015). Multiple comparisons between different genotypes were carried out by Tukey HSD, and significance level was set at *p* < 0.05. The variance explained by the SNP $\left(\sigma_{\text{SNP}}^{2}\right)$ was estimated for each SNP‐trait test as follows:

$$\sigma_{\text{SNP}}^{2}=100\times \frac{\left(\text{RMS - FMS}\right)}{\text{RMS}}$$

where RMS is the residual of the reduced model (SNP effect excluded), and FMS is the residual of the full model (SNP effect included) (Gorshkova et al., 2006).

## 3 RESULTS

### 3.1 Polymorphisms in coding region *KLF3* gene

**FIGURE 1:**
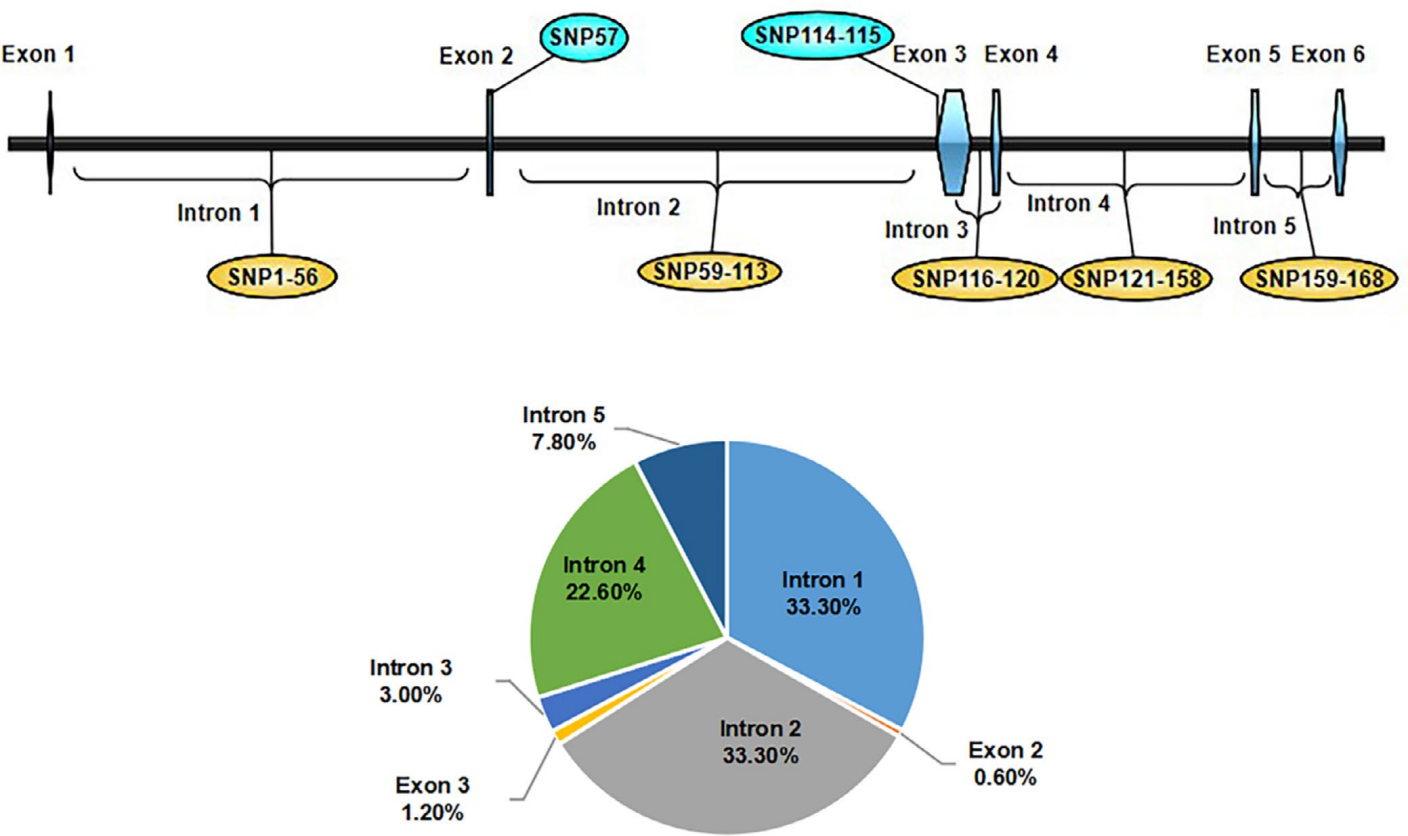
The distribution of SNPs in coding region of *KLF3*

A total of 168 SNPs were detected in coding region of *KLF3*. Among them, 165 SNPs were located in introns, and three SNPs were located in exons. The distribution of SNPs on *KLF3* was shown in the Figure 1 (Table S1). Totally, 329 male birds from 19th generation (G19) were genotyped for these SNPs by sequencing.

### 3.2 Association analysis between SNPs and abdominal fat traits

We carried out the correlation analysis between the 168 SNPs and abdominal fat traits. The results were shown in Table 1. Totally, three SNPs (g.3452T > C in intron 2, g.8663A > G in intron 4 and g.10751G > A in intron 4) were significantly associated with AFW and AFP (FDR < 0.05). Percentage of additive genetic variance explained (PVE) by these three SNPs varied from 2.01% to 8.81%. Multiple comparisons between their different genotypes were shown in Table 1. Birds with homozygous wild genotype were significantly lower than the other two genotype individuals for two SNPs (g.3452T > C, g.8663A > G) in AFW and AFP (*p* < 0.05). Birds with homozygous mutation genotypes had lower AFW and AFP for g.10751G > A (*p* < 0.05).

**TABLE 1:** Correlation analysis of three SNPs of *KLF3* with AFW and AFP (Least square means ± standard error, LSM ± *SE*)

SNPs	Traits	Least square means ± standard error (LSM ± *SE*)			FDR	PVE (%)
g.3452T > C		T/T	T/C	C/C		
	AFW (g)	56.320 ± 0.817^a‐c^	53.650 ± 1.329^a‐c^	66.396 ± 3.032^a‐c^	0.0315*	2.01
	AFP (%)	2.917 ± 0.040^a‐c^	2.735 ± 0.065^a‐c^	3.479 ± 0.146^a‐c^	0.0005*	4.95
g.8663A > G		A/A	A/G	G/G		
	AFW (g)	56.736 ± 0.807^a‐c^	51.559 ± 1.629^a‐c^	64.639 ± 3.972^a‐c^	0.0315*	2.20
	AFP (%)	2.932 ± 0.040^a‐c^	2.654 ± 0.080^a‐c^	3.349 ± 0.194^a‐c^	0.0053*	2.64
g.10751G > A		G/G	G/A	A/A		
	AFW (g)	68.376 ± 3.231^a‐c^	55.981 ± 1.218^a‐c^	55.566 ± 0.832^a‐c^	0.0315*	7.15
	AFP (%)	3.546 ± 0.157^a‐c^	2.867 ± 0.060^a‐c^	2.878 ± 0.042^a‐c^	0.0053*	8.81

PVE, Percentage of additive genetic variance explained by the SNPs.

^a‐c^ LSMs within a column with no common superscript differ significantly (*p* < 0.05).

* FDR < 0.05.

The genotype frequencies and allele frequencies of the three SNPs (g.3452T > C, g.8663A > G and g.10751G > A) of NEAUHLF were analysed, the results indicated that the allele frequencies of the three SNPs were extremely significantly different between the lean and fat lines (*p* < 0.01). Moreover, polymorphism information content (PIC) indicated that the g.3452T > C, g.8663A > G and g.10751G > A were lowly polymorphic in the lean line, and moderately polymorphic in the fat line (Table 2).

**TABLE 2:** Genotype, allele frequencies and genetic diversity parameters of three SNPs in lean and fat lines

SNPs	Lines	Genotype frequencies (No. individuals)			Allele frequencies		*χ* ^2^	Ho	He	Ne	PIC
g.3452T > C		T/T	T/C	C/C	T	C					
	Lean	0.847 (144)	0.141 (24)	0.012 (2)	0.918	0.082	19.32	0.849	0.151	1.177	0.139
	Fat	0.660 (105)	0.277 (44)	0.063 (10)	0.799	0.201	*p* < 0.0001	0.679	0.321	1.473	0.270
g.8663A > G		A/A	A/G	G/G	A	G					
	Lean	1 (170)	0 (0)	0 (0)	1	0	74.49	1	0	1	0
	Fat	0.641 (102)	0.320 (51)	0.038 (6)	0.802	0.198	*p* < 0.0001	0.682	0.318	1.465	0.267
g.10751G > A		G/G	G/A	A/A	G	A					
	Lean	0.012 (2)	0.194 (33)	0.794 (135)	0.109	0.891	11.46	0.806	0.194	1.241	0.175
	Fat	0.044 (7)	0.321 (51)	0.635 (101)	0.204	0.796	*p* < 0.0001	0.675	0.325	1.481	0.272

### 3.3 Dual luciferase report gene assay

**FIGURE 2:**
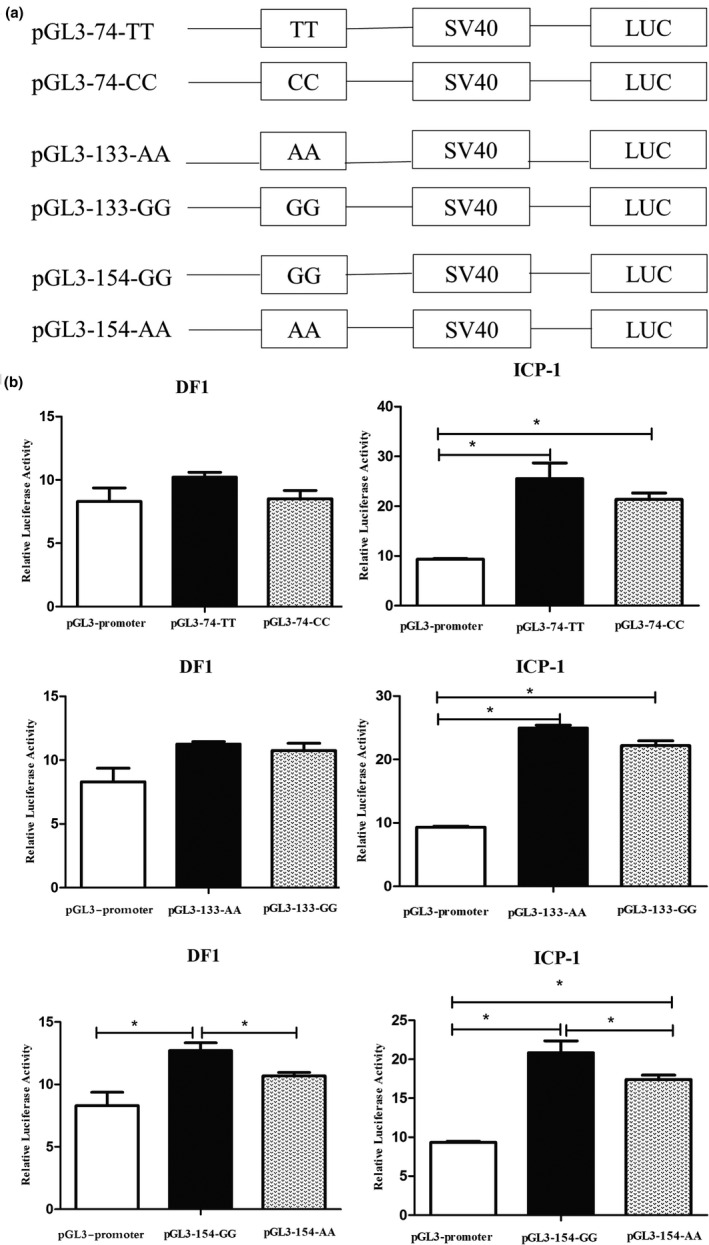
Promoter transcriptional activity affected by candidate SNPs. (a) Schematic presentation of luciferase reporter constructs. Genomic fragments carrying the SNP were cloned upstream of the SV40 promoter of the pGL3 vector. (b) Luciferase assays in DF1 and ICP‐1 cells. *p < 0.05

In order to identify functionality of three SNPs (g.3452T > C, g.8663A > G and g.10751G > A) for abdominal fat in chickens, we inserted the DNA fragment containing their different alleles into the upstream of the pGL3‐promoter SV40 vector, and constructed the dual luciferase reporter vectors (Figure 2a), and determined activity of different alleles in both in ICP‐1 and DF1 cells. The results showed that the g.10751G > A luciferase activity of different alleles was significantly different in both ICP‐1 and DF1 cells, and the luciferase activity of allele G was significantly higher than that of allele A (*p* < 0.05). For the g.3452T > C and g.8663A > G, luciferase activity of different alleles did not exhibit significant differences (Figure 2b).

### 3.4 Bioinformatics analysis

In order to investigate potential molecular mechanism underlying the association of abdominal fat content with chicken *KLF3* g.10751G > A, we carried out in silico analysis of the transcription factor binding site of *KLF3*. The results showed that g.10751G > A was located in multiple transcription factor binding regions (Table 3).

**TABLE 3:** Bioinformatics analysis of the g.10751G > A

Mutation Site	Base Group	Transcription factors	Function	References
g.10751G > A	G	CREB1 OCT1 AP1 P53 SP1	Associated with lipid storage or fat cell regulation To play a role in glucose and lipid metabolism in liver To affect adipocyte differentiation by regulating PPARγ Related to adipose tissue metabolism and homeostasis To promote fat production	Xu et al., 2017 Chen et al., 2014 Luther et al., 2014 Krstic et al., 2018 Lu et al., 2010
	A	GATA2	To increase adipose tissue differentiation	Menghini et al., 2005

## 4 DISCUSSION

Excessive abdominal fat deposition in broilers not only reduces reproductive performance and causes metabolic diseases but also reduces meat quality (Chen et al., 2019). Under this circumstance, molecular marker‐assisted selection (MAS) is an efficient approach to enhance selection efficiency and further improve production performance.

Previous studies have shown that several members of the KLF family play a vital role in mammalian fat deposit (Farmer, 2006; Lefterova & Lazar, 2009; Rosen & MacDougald, 2006). Kruppel‐like factor 3 (KLF3) is an important regulator of fatty acid synthesis, lipid secretion and degradation, which are critical in mammalian lipid metabolism (Hashmi et al., 2011; Zhang et al., 2009, 2011, 2013). Researches showed that *KLF3* also plays an important role in adipogenesis and lipid metabolism in agricultural animals (Guo, Raza, et al., 2018; Pertille et al., 2015). Our previous study indicated that *KLF3* was expressed ubiquitously in chicken tissues and expressed consecutively during the chicken adipose tissue development from 1 to 12 weeks of age. In addition, the relative *KLF3* mRNA expression levels of lean males were higher at 7 weeks of age and lower at 10 weeks of age than those of fat males, suggesting that the difference in *KLF3* expression in abdominal adipose tissue might contribute to the fatness trait difference between fat and lean birds (Zhang et al., 2014). Therefore, in the present study, the *KLF3* was selected as a candidate gene to investigate associations of gene polymorphisms with chicken abdominal fat in NEAUHLF and functional variants in vitro.

Among 168 SNPs detected in coding region of *KLF3*, three SNPs (g.3452T > C, g.8663A > G and g.10751G > A) were highlighted significantly associated with abdominal fat traits (AFW and AFP, FDR < 0.05). As far as application is concerned, homozygous wild genotype for g.3452T > C, g.8663A > G and homozygous mutated genotypes for g.10751G > A are favourable in that the birds with these genotypes have lower AFW or AFP. In addition, there were significant differences between the two lines in allele frequencies of these three SNPs (Table 2) (*p* < 0.01). Generally speaking, artificial selection for important traits of domesticated animals is accompanied with alteration of allele frequencies of gene, frequencies of the alleles favourable for human are more likely to be increased, whereas unfavourable alleles tend to be decreased (Zhou et al., 2020). NEAUHLF used in the present study has been divergently selected for AFP since 1996, exhibiting significant differences in AFP after 4th generation. It is expected that changing trend of alleles affecting AFP is similar to that of AFP. Therefore, three SNPs (g.3452T > C, g.8663A > G and g.10751G > A) are likely subjected to selection with abdominal fat due to extremely significant differences in allele frequencies between fat and lean lines. Regretfully, however, it is only in 19th generation that differences in alleles between two lines were investigated in this study. It is probably more reasonable to probe changing trend of alleles of these three SNPs between fat and lean lines in multiple and consecutive generations.

In addition to association analysis, we also provided functional evidence supporting a role for the SNPs in abdominal fat deposition. Studies have found that introns can affect gene transcription regulation, and intron mutations may change gene transcription efficiency (Beohar & Kawamoto, 1998; Do et al., 2010; Meyer et al., 2008). For instance, the C > T mutation in intron 1 of human CCNE1 changes the transcription efficiency of CCNE1 by changing the affinity between CCNE1 and transcription factor KLF5 (Pattison et al., 2016). C > T mutation in intron 2 of Kras in mice changes the transcription efficiency of Kras by changing the binding ability of Kras and transcription factor NF‐Y (Gorshkova et al., 2006). We performed luciferase reporter assays to investigate whether these three intronic SNPs (g.3452T > C, g.8663A > G and g.10751G > A) affected transcriptional activity. In the chicken DF1 and ICP‐1 cell, the luciferase activity of allele G was significantly higher than that of allele A of the g.10751G > A. There was no significant difference in luciferase activity between different alleles at other SNPs (g.3452T > C, g.8663A > G). These results indicated that g.10751G > A was a functional variant affecting *KLF3* transcriptional efficiency in vitro.

Studies have reported that the binding of transcription factors to intronic regions is modulated by intronic SNPs within transcription factor binding sites (Tsukada et al., 2006). In silico analysis suggested that the g.10751G > A located in multiple transcription factor binding regions, which may affect the transcription efficiency of the *KLF3* by binding to some transcription factors (Table 3). CREB1 has the function in lipid storage or fat cell regulation (Xu et al., 2017). It has been reported that AP1 can affect adipocyte differentiation by regulating PPARγ (Luther et al., 2014). Transcription factor OCT1 is highly expressed in mouse liver and plays a major role in glucose and lipid metabolism in liver (Chen et al., 2014). P53 functions in adipose tissue metabolism and homeostasis (Krstic et al., 2018). In cancer cells, Sp1 promotes fat production by up‐regulating the expression of sterol regulatory element binding protein‐1c (srebp‐1c) and FASN (Lu & Archer, 2010). Phosphorylation of GATA2 by Akt increases adipose tissue differentiation and reduces adipose tissue‐related inflammation (Menghini et al., 2005). Based on close relationship of transcription factors CREB1, AP1, OCT1, P53, Sp1 and GATA2 with fat deposit, we speculate that g.10751G > A could change the gene transcription efficiency through the binding ability of a certain (some) transcription factor (factors) to *KLF3*. However, whether the predicted transcription factors directly bind to the promoter region of chicken *KLF3* still needs to be further verified by EMSA and ChIP experiments.

## 5 CONCLUSIONS

Three SNPs in *KLF3*, including g.3452T > C, g.8663A > G and g.10751G > A, may be important molecular markers that affect chicken abdominal fat traits. Taking into consideration that its strongest association with AFW (PVE = 7.15%) and AFP (PVE = 8.81%), and significant differences in luciferase activity of different alleles in vitro, g.10751G > A is considered as a functional SNP for abdominal fat in chickens. These findings will provide useful molecular markers for genetic improvement of abdominal fat content in chicken MAS programme.

## CONFLICT OF INTEREST

The authors declare that there are no conflicts of interest.

## AUTHOR CONTRIBUTIONS

**Weijia Wang:** Conceptualization; Formal analysis; Writing‐original draft; Writing‐review & editing. **Yudong Li:** Software; Writing‐review & editing. **Ziwei Li:** Methodology; Writing‐review & editing. **Ning Wang:** Writing‐review & editing. **Fan Xiao:** Writing‐review & editing. **Haihe Gao:** Writing‐review & editing. **Huaishun Guo:** Writing‐review & editing. **Hui Li:** Writing‐review & editing. **Shouzhi Wang:** Conceptualization; Writing‐original draft; Writing‐review & editing.

## ETHICAL APPROVAL

All animal work was conducted according to the guidelines for the Care and Use of Experimental Animals established by the Ministry of Science and Technology of the People's Republic of China (approval number: 2006‐398), and was approved by the Laboratory Animal Management Committee of Northeast Agricultural University.

## Supporting information

Table S1
